# Quality Control on Herbal Medicine and Its Application

**DOI:** 10.1155/2018/3294398

**Published:** 2018-03-15

**Authors:** Gallant Kar-Lun Chan, Rentian Wu, Vicky Ping Chen, Kevin Yue Zhu, Yingqing Du

**Affiliations:** ^1^Division of Life Science and Center for Chinese Medicine, The Hong Kong University of Science and Technology, Clear Water Bay Road, Clear Water Bay, Hong Kong; ^2^Department of Molecular Pharmacology and Experimental Therapeutics, Mayo Clinic, Rochester, MN 55905, USA; ^3^Jiangsu Key Laboratory for High Technology Research of TCM Formulae and Jiangsu Collaborative Innovation Center of Chinese Medicinal Resources Industrialization, State Key Laboratory Cultivation Base for TCM Quality and Efficacy, Nanjing University of Chinese Medicine, Nanjing, Jiangsu, China; ^4^Hanshan Normal University, Chaozhou, Guangdong 521041, China

In this issue, you will see how researchers applied systematic biological method to illustrate the importance of genuineness, so-called “Daodi,” of herbal medicinal material. Metabolomics study on herbal medicine as well as their interactions with commonly used compound medicine, such as Aspirin, will also be demonstrated. Curcumin was known to be a chemical compound with many strong biological functions. We will show you an advanced extraction methods which helps establishing quality control standards for herbal medicine with Curcumin. Moreover, the detailed descriptions of clinical effect and safety of herbal medicinal formulation will be covered in this issue. Last but not least, a fast and innovative drug screening platform, namely HerboChips, will be introduced. The paper describes how HerboChips makes drug screening down to molecular level from herbal medicine become feasible.

Although the background of the authors varies, they share one goal—to unmask the beauty of herbal medicine, a system which support our health and population for more than several thousands of years in human history.

